# Editorial: Psychological status of medical workers throughout the COVID-19 pandemic and beyond: Mental health emergence, prevalence and interventions

**DOI:** 10.3389/fpubh.2023.1186807

**Published:** 2023-03-27

**Authors:** Lawrence T. Lam, Prasuna Reddy

**Affiliations:** ^1^Faculty of Medicine, Macau University of Science and Technology, Macau, Macao SAR, China; ^2^Faculty of Health, University of Technology Sydney, Sydney, NSW, Australia; ^3^Faculty of Medicine and Health, University of Sydney, Sydney, NSW, Australia; ^4^Faculty of Medicine and Health Sciences, Macquarie University, Sydney, NSW, Australia

**Keywords:** COVID-19, mental health, healthcare worker, epidemiology, intervention

COVID-19, the first documented coronavirus pandemic in history has been considered a human catastrophe unseen in the past century (1). The impact of the pandemic is tremendous, in terms of mortalities, long-term morbidities, and the global economy. The World Health Organisation (WHO) has estimated that COVID-19 pandemic-related deaths, recognized as “excess deaths,” to be 14.9 million (95% C.I. =13.3–16.6 million) in 2020 and 2021 (2). Excess deaths refer to the difference between the number of deaths that have occurred and the expected number of deaths, based on previous data, in the absence of the pandemic (2). In terms of the impact of the COVID-19 pandemic on the global economy, it has been recognized by the World Bank that the largest worldwide economic crisis in more than a century has been triggered (3). Based on the available data since the onset of the pandemic, the Work Bank notes that many governments have made decisive economic policy responses that are successful in mitigating the impact of the pandemic on the national and international economic crisis in the short term (3). However, these immediate and drastic responses of economic reliving packages would have longer-term consequences in creating more debts, particularly among countries of emerging economies. This will, in turn, create significant global inequality and poverty within and across many countries (3). Such phenomena have been demonstrated in many studies on loss of income and unemployment during the pandemic (4).

The pandemic has had a direct impact on the physical aspects of health, and impinges on the mental health, of our worldwide population. As people are exposed to traumatic events, frontline medical and health professionals have been the group that crops the hardest hit (5). It has long been recognized that frontline healthcare workers, including medical, nursing, and allied health professionals, are at high risk of mental health problems due to frequent exposure to traumatic events (6). However, the scenario of a worldwide pandemic and its impact on the healthcare system is unprecedented. The psychological and mental health sequelae of healthcare workers to such a global catastrophe, which has never been seen and experienced in the current generation before, warrant thorough documentation. The effort and wisdom of various intervention programs put in place by different jurisdictions to assist frontline workers in mitigating and alleviating the burden of psychological and emotional trauma are certainly worth noting and reporting.

This Research Topic aims to report the work of a group of researchers who have been investigating the issue of the psychological status and mental health problems of healthcare workers during the pandemic from diverse disciplinary and methodological backgrounds.

This series consists of 16 articles of different study designs reporting on the different ways the pandemic impinged the mental health of frontline healthcare workers, including medical, nursing, and allied health professionals. Of these, ten were cross-sectional surveys using self-reported questionnaires (El Sharif et al.; Li et al.; Liu et al.; Mei et al.; Ning et al.; Peng J. et al.; Peng P. et al.; Pahrol et al.; de Vroege and van den Broek; Zhao et al.). Four studies employed a qualitative approach with semi-structured interviews with participants (Alsaeed et al.; Ding et al.; Mediavilla et al.; Tan Cheung et al.). Banse et al. reported a case study and Xu et al. followed a cohort of hospital staff who had been involved in the Employee Assistance Program (EAP).

For geographical distribution of these studies, since the first outbreak of the COVID-19 pandemic occurred in China, it is not surprising to find that more than half of these studies were conducted in China, including Hong Kong the Special Administrative Region (Ding et al.; Li et al.; Liu et al.; Mei et al.; Ning et al.; Peng P. et al.; Tan Cheung et al.; Xu et al.; Zhao et al.). Four studies were conducted in Europe, including the UK (Peng J. et al.), the Netherlands (de Vroege and van den Broek), Belgium (Banse et al.), and Spain (Mediavilla et al.). Two reported findings are from the Middle East with one from Palestine (El Sharif et al.) and a recent one from Kuwait (Alsaeed et al.).  Pahrol et al. investigated the topic in Malaysia.

To explore the emerging mental health problems among frontline healthcare workers, a few qualitative studies explored the issue with medical and nursing staff mainly in the hospitals where they were exposed to patients with greater severity. Tan Cheung et al. found that interviewed nurses were intensely fearful, worried, and anxious, They were worn out, and distressed with their psychosocial and physical health greatly impacted. They were also found to have limited ways of coping with distress. In the Spanish study by Mediavilla et al., it was found that healthcare workers were psychologically and morally distressed. Moreover, the mental health strategies implemented in the hospital did not fully address the needs of healthcare workers.

In terms of the prevalence of mental health problems exhibited during the pandemic period between early 2020 and the end of 2022, various studies provided slightly different estimates. Mei et al. reported that nearly 11% of frontline medical staff had exhibited PTSD symptoms during the first outbreak of COVID in Wuhan. It was also found that insomnia mediated the association between stress and PTSD and compassion moderated the relationship. On the other hand, de Vroege and van den Broek found that about 50% of respondents had experienced stress, anxiety, anger, and sadness with 4% of healthcare workers of mental healthcare institutions considered resigning. Another survey in China by Ning et al. revealed that nearly 24% of the medical and nursing staff involved in the study had symptoms of depression, 27% anxiety, and 16% stress. Moreover, medical staff has a higher rate of depression and anxiety than nurses. Pahrol et al. studied healthcare workers in Malaysia and found that about 19% of respondents showed symptoms of PTSD, however, the majority (92%) perceived the outbreak has a low impact on their life and work.

Some risk and protective factors were identified in these studies. For example, the study by Li et al. among dentists in China found that various situational variables, such as the impact of COVID on daily life and work, exposure to the virus, and lack of awareness of the preventive and control measures, were associated with mental health problems. Ning et al. also identified that the perceived risk of exposure was associated with both depression and anxiety. The UK study by Peng J et al. discovered that married women had lower mental well-being than married men and the well-being of single women was significantly lower than that of married women and men. On the other hand, environmental and organizational factors would be protective of healthcare workers' mental health. El Sharif et al.'s study in Palestine found that better mental health was associated with confidence in the system's ability to manage the pandemic. Furthermore, training in IPC procedures and sufficient provision of PPE increased the trust of staff.

For the intervention and prevention of mental health problems among healthcare workers during and beyond the pandemic period, some information and examples have been provided from a few studies. In the study by Alsaeed et al. among Keratinase healthcare workers, three main themes emerged on the readiness of healthcare workers for future crises. These included the enhancement of self-resilience, a better-equipped workforce and healthcare environment, mitigation of stigma, and increased public awareness of preventive measures. Banse et al. also showcased a multiple-approach intervention program implemented in a Belgium hospital to support workers during COVID and the process of reinforcing the impact of the program in preparation for future similar crises. Xu et al. reported the results of an intervention program using the EAP as a means to provide support to hospital staff. Results indicated significant reductions in mental health problems, including depression and anxiety, among staff after they completed the EPA program.

We hope this series of articles can draw attention to the issue of mental health and mental well-being of all personnel, particularly frontline healthcare workers, after being exposed to traumatic events and global health crises, such as the COVID-19 pandemic. The experience of these trauma exposures could be long-lasting and affected individuals may require lengthy rehabilitation. More thorough research into effective preventive intervention strategies is needed to enhance the readiness and preparedness of all walks of life to face up the challenges of similar global crises in the future.

## Author contributions

All authors listed have made a substantial, direct, and intellectual contribution to the work and approved it for publication.
